# Pathological Cluster Identification by Unsupervised Analysis in 3,822 UK Biobank Cardiac MRIs

**DOI:** 10.3389/fcvm.2020.539788

**Published:** 2020-11-16

**Authors:** Qiao Zheng, Hervé Delingette, Kenneth Fung, Steffen E. Petersen, Nicholas Ayache

**Affiliations:** ^1^Université Côte d'Azur, Inria, Sophia Antipolis, Valbonne, France; ^2^National Institute for Health Research Barts Biomedical Research Centre, William Harvey Research Institute, Queen Mary University of London, London, United Kingdom; ^3^Barts Heart Centre, St Bartholomew's Hospital, Barts Health National Health Service Trust, London, United Kingdom

**Keywords:** cluster analysis, feature extraction, cine MRI, UK Biobank, cardiac pathology

## Abstract

We perform unsupervised analysis of image-derived shape and motion features extracted from 3,822 cardiac Magnetic resonance imaging (MRIs) of the UK Biobank. First, with a feature extraction method previously published based on deep learning models, we extract from each case 9 feature values characterizing both the cardiac shape and motion. Second, a feature selection is performed to remove highly correlated feature pairs. Third, clustering is carried out using a Gaussian mixture model on the selected features. After analysis, we identify 2 small clusters that probably correspond to 2 pathological categories. Further confirmation using a trained classification model and dimensionality reduction tools is carried out to support this finding. Moreover, we examine the differences between the other large clusters and compare our measures with the ground truth.

## 1. Introduction

In recent years, more and more data are made accessible for research in medical image analysis. For instance, the UK Biobank study by Petersen et al. (1) has released a dataset containing the cardiac cine MRI images of thousands of volunteers, from which various key cardiovascular functional indexes can be extracted for analysis (2). The Alzheimer's Diseases Neuroimaging Initiative [ADNI (3)] has accumulated brain scan images of about 2,000 participants. The abundant data available in the community are certainly a highly valuable resource (4, 5). Researchers are hence less constrained by the scarcity of data, which has been a prevailing challenge for a long time. Further research is necessary (6, 7) on new topics associated with big data. For example, one major challenge is how to make good use of unlabeled data (8, 9). In fact, while there are more and more labeled data available, an important part of medical images is still unlabeled. This is understandable as it is in general expensive and tedious to diagnose and label cases by human experts. Methods that can extract useful information from unlabeled data are hence interesting and might potentially save a lot of time and effort.

Many research projects have been developed to perform pathology-related analysis using features extracted from medical images. Many of these works focus on brain scan images. For example, in (10), feature vectors extracted from brain images are used for the prediction of autism spectrum disorder and Alzheimer's disease. An anatomical landmark-based deep feature representation for MRI is proposed in (11) for diagnosis of brain disease. Some other studies are based on digital histopathological images. For instance, Madabhushi and Lee (12) discuss the predictive modeling of digital histopathological images from a detection, segmentation, feature extraction, and tissue classification perspective. Komura and Ishikawa (13) review the machine learning methods for histopathological image analysis. But there are less pathology-related and feature-based research on cardiac images than on brain scan images and digital histopathological images. And currently, this research (14–19) is mostly about pathology classification in the dataset of Automatic Cardiac Diagnosis Challenge (ACDC) of MICCAI 2017, which contains 100 cases with labels. The work of Attar et al. (2) is one of the very first projects to propose a fully automatic, high-throughput image parsing workflow for the analysis of cardiac MRI in UK Biobank with systematic tests of the performance. In addition to MRI, echocardiography sequences are also useful in characterizing cardiac pathology (20). As an extension of the previous works and a challenge to ourselves, we wish to conduct unsupervised analysis on large unlabeled cardiac image datasets.

Clustering, an unsupervised machine learning technique that groups similar entities together, might be suitable for analyzing large unlabeled datasets. Up to now, clustering has been widely used on image segmentation in medical image analysis. For example, Kinani et al. (21) develop a tool based on clustering to outline brain lesion contours. Unsupervised segmentation of 3D lung Computed tomography (CT) images is proposed in (22) based on clustering and deep representation learning. Some studies show that clustering is also a powerful tool for classification. For instance, a clustering method is applied to classify the analyzed brain images into healthy and multiple sclerosis disease in (23). Kawadiwale and Rane (24) introduce various clustering techniques to classify brain Magnetic resonance (MR) images into normal and malformed. While most of the application of clustering in the domain is on brain images, we aim to extend its application to cardiac images. Furthermore, we consider clustering as an example of the family of unsupervised learning methods. As pointed out above, how to extract useful information from unlabeled data of medical images is an important research topic, and unsupervised learning methods are natural candidates for this task. We hence hope that the results obtained with clustering would inspire and encourage researchers to further consider the family of unsupervised learning methods.

**Figure 1:**
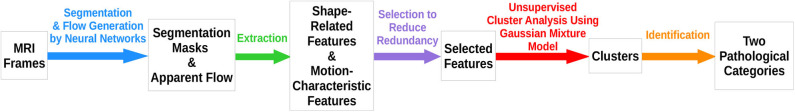
Graphical abstract of the analysis performed in this study.

In this paper, we perform a cluster analysis of a group of features extracted from the cardiac MR images of the UK Biobank dataset. The process of analysis is summarized in Figure 1. With neural networks trained to perform segmentation and flow generation on MRI frames (14, 25), segmentation masks and apparent flow are generated for the extraction of several features. After feature selection to reduce information redundancy, unsupervised cluster analysis using Gaussian mixture model is carried out to give rise to clusters, among which 2 are identified as probably corresponding to pathological categories. We hence demonstrate that given a large dataset, even with a small number of features that contain only a very limited amount of information available in cardiac MR images, unsupervised analysis enables us to come up with valuable results.

Our main contributions are three-fold:

- We conduct a cardiac pathology–related analysis on a large unlabeled dataset.
- As a novel application of a classic method in medical image analysis, clustering is used in our analysis to group cases without supervision.
- Among the resulting clusters, 2 can indeed be identified as leaning toward pathological categories.

## 2. Data

### 2.1. UK Biobank

The proposed method was applied to the very large UK Biobank cardiac MRI dataset [see (26)^1^]. It comprises short-axis balanced steady-state free precession (bSSFP) cine MRI of about 5,000 participants from the general population, stored in DICOM image files. More details of the magnetic resonance protocol are available in (26). Each time series consists of 3D volumes with slice thickness of 8 mm for short-axis images. The in-plane resolution is 1.8 × 1.8 mm. Volumes at end-diastole (ED) and end-systole (ES) and ejection fraction for left ventricle (LV) cavity were derived from InlineVF analysis algorithm (27, 28) performed by UK Biobank (Field 22421-22422). Those values are considered in this paper as ground-truth (or reference) values. To be consistent with our previous research, such as in (25) and (14), we exclude roughly 1,000 cases that are provided with incomplete or unconvincing ground truth. The remaining 3,822 cases are then used for cluster analysis. For part of these cases, the measures of LV volumes at ED and ES and LV ejection fraction are provided as ground truth by UK Biobank.

As pointed out on the website of UK Biobank^2^ and in (29), while UK Biobank participants are not representative of the general population with evidence of a “healthy volunteer” selection bias (and hence cannot be used to provide representative disease prevalence and incidence rates), valid assessment of exposure–disease relationships are nonetheless widely generalizable and does not require participants to be representative of the population at large.

### 2.2. Automatic Cardiac Diagnosis Challenge

In the experiment part, we will show the correspondence between some resulting clusters and the definition of some pathology categories defined in the ACDC. Furthermore, a classification model trained on ACDC by (14) will be applied on UK Biobank for comparison with the clustering method proposed in this paper. The ACDC dataset^3^ consists of 100 cases, which are divided into the following 5 pathological groups of equal size according to their pathology on either the LV or the right ventricle (RV):

- Dilated cardiomyopathy (DCM): LV cavity volume at ED larger than 100 *mL*/*m*^2^ and LV ejection fraction lower than 40%;
- Hypertrophic cardiomyopathy (HCM): LV cardiac mass higher than 110 *g*/*m*^2^, several myocardial segments with a thickness higher than 15 mm at ED and a normal ejection fraction;
- Myocardial infarction (MINF): LV ejection fraction lower than 40% and several myocardial segments with abnormal contraction;
- RV abnormality (RVA): RV cavity volume higher than 110 *mL*/*m*^2^ or RV ejection fraction lower than 40%;
- Normal subjects (NOR).

The definitions of the pathological groups above might seem somewhat simplistic. For example, guidelines for cardiologist encompass more detailed criteria for diagnosing HCM. But these more operative and straightforward definitions are good enough for the current study to show the effect of the proposed methods.

## 3. Methods

There are mainly three steps in the proposed method: feature extraction, feature selection, and cluster analysis.

### 3.1. Feature Extraction

The feature extraction method used in this paper is the same as the one proposed in our previous work published by Zheng et al. (14). We briefly describe its principal steps again as follows.

The first part of the feature extraction method generates 7 shape-related features. Segmentation with spatial propagation has been proven to be consistent and robust (25, 30, 31). On the one hand, spatial propagation enforces the consistency of segmentation across different slices, including the most challenging ones. On the other hand, preprocessing techniques such as extreme pixel value cutting, resizing, and normalization are applied to minimize the differences across subjects and datasets such that the method can be successfully applied regardless of sites and scanners. With the cardiac segmentation method proposed in (25), the cardiac images are segmented such that we obtain the masks of LV, left ventricle myocardium (LVM), and RV on both ED and ES frames. Compared to single ventricle segmentation models, a bi-ventricular model like that in (25) not only is faster since it goes through each image once instead of twice, but also might be more accurate and robust as it may exploit the interrelationship between the 2 ventricles. Then the volumes of LV, LVM, and RV at both ED and ES can be computed directly, as can the thickness of LVM. Finally, 7 shape-related features are generated (the first 7 terms in Table 1).

**Table 1:** The 9 features generated by our feature extraction method.

**Feature**	**Notion**	**Selected**
RV volume at ED	*V*_*RV, ED*_	Yes
LV volume at ES	*V*_*LV, ES*_	Yes
RV ejection fraction	*EF*_*RV*_	Yes
LV ejection fraction	*EF*_*LV*_	No
Ratio between RV and LV volumes at ED	*R*_*RVLV, ED*_	Yes
Ratio between LVM and LV volumes at ED	*R*_*LVMLV, ED*_	Yes
Maximal LVM thickness in all the slices at ED	*MT*_*LVM, ED*_	Yes
Radius motion disparity	*RMD*	Yes
Thickness motion disparity	*TMD*	Yes

*Among them 8 are selected for cluster analysis*.

The second part of the method extracts 2 motion-characteristic features. Using a neural network that outputs apparent flow maps given image pairs, we get a series of apparent flow maps characterizing the in-plane motion for each MRI slice of each case. Combined with the LVM segmentation mask obtained as described above, the motion of each myocardium pixel is hence available. Eventually, 2 features are computed to present the disparity of the radial myocardial motion and the myocardial thickening, respectively (the last 2 rows in Table 1).

In total, from the images of each case, 9 features characterizing the shape and the motion of the heart are extracted.

### 3.2. Feature Selection

As shown in (14), these extracted features can be used for cardiac pathology classification in the ACDC dataset with performances comparable to the state-of-the-art. However, these features are not necessarily independent. Some might be redundant if there are highly correlated feature pairs. In cluster analysis, if too many variables are used simultaneously, the redundant ones serve only to create noise that harms the clustering. So it is helpful to select a sub-group of features by removing highly correlated feature pairs.

For each pair among the 9 extracted features, we compute the Pearson correlation coefficient (i.e., Pearson's r) and the maximal information coefficient (MIC) (32). The former measures the linear correlation between 2 features, whereas the latter measures the mutual information between features. If there is any highly correlated pair according to these measures (i.e., Pearson correlation coefficient of absolute value above 0.8, or MIC above 0.5), we will exclude 1 feature in this pair. The remaining features are then considered as selected.

### 3.3. Cluster Analysis

Cluster analysis is the task of grouping objects such that objects in the same group (also called cluster) are more similar to each other than to those in other groups. Some common clustering methods are agglomerative hierarchical clustering, k-means clustering, and Gaussian mixture model clustering. In this study, we perform a model selection of Gaussian mixture model using the Bayesian information criterion (BIC). Then the selected Gaussian mixture model is applied to cluster the 8 selected features.

#### 3.3.1. Gaussian Mixture Model Selection

A Gaussian mixture model (33) is a probabilistic model that assumes that the data points are generated from a mixture of a certain number of Gaussian distributions with unknown parameters. An expectation–maximization algorithm is used to iteratively estimate its parameters from data. Then the fitted model can assign to each sample the Gaussian component it most likely belongs to.

We use the Gaussian mixture model as implemented in scikit-learn (34). It has 2 major parameters, the type of covariance matrix and the number of components, upon which a selection is necessary. For this purpose, we calculate the BIC (35) for Gaussian mixture models with different types of covariance matrix and numbers of components. In theory, BIC recovers the true number of components approximately. We fit the Gaussian mixture models with the following types of covariance matrix:

- “tied”: all components share the same covariance matrix;
- “diag”: each component has its own diagonal covariance matrix;
- “full”: each component has its own covariance matrix.

The number of components is also varied. By looking for models with the smallest BIC scores, we wish to select the most simple model that can fit the data, thereby identifying the most suitable type of covariance matrix and a range of reasonable numbers of components.

The number of components will finally be determined by examining the sizes of resulting clusters of the Gaussian mixture models. More details will be provided in section 4.

#### 3.3.2. Analysis of the Resulting Clusters

The clusters generated by the selected model will be examined. In particular, we verify if the cases in any of the clusters correspond to a pathological category according to the definitions of pathologies given by the ACDC.

## 4. Experiments and Results

### 4.1. Feature Extraction

With the feature extraction method introduced in section 3, for each of the 3,822 UK Biobank cases, 9 feature values are extracted.

### 4.2. Feature Selection

**Figure 2:**
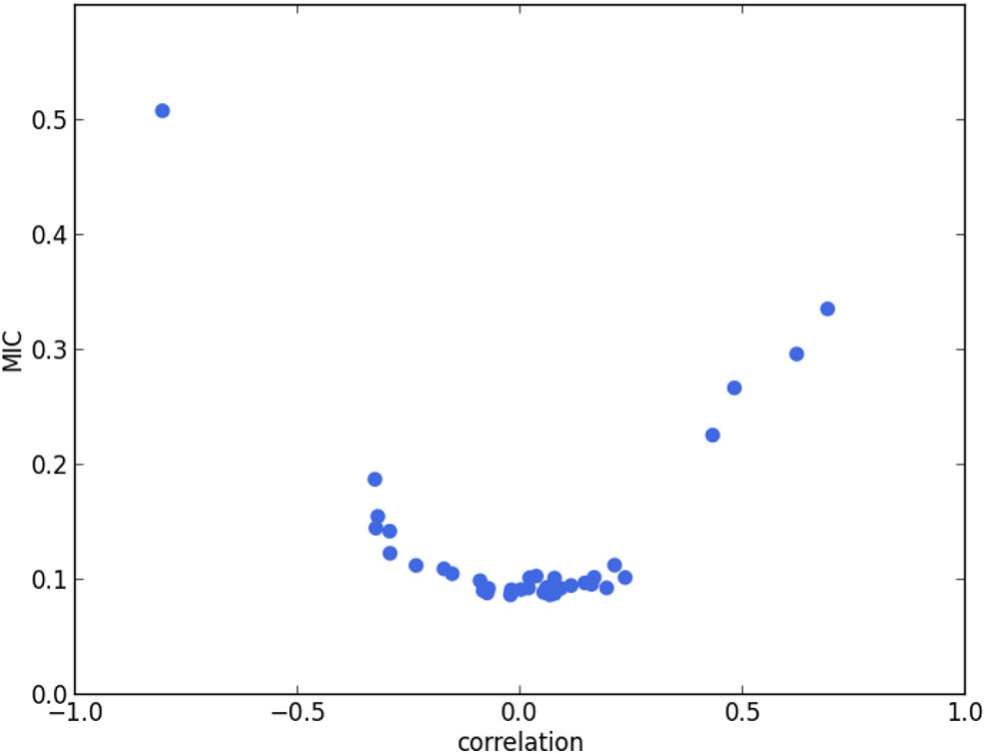
Pearson correlation coefficient (i.e., Pearson's r) vs. MIC. Each point corresponds to a pair of features. The point in the upper left corner corresponds to *V*_*LV, ES*_ and *EF*_*LV*_. The strong negative correlation between these 2 features is reasonable, since by definition *EF*_*LV*_ = 1−*V*_*LV, ES*_/*V*_*LV, ED*_, in which *V*_*LV, ED*_ is the LV volume at ED.

We calculate the Pearson correlation coefficient and MIC for each pair of features among the 9 extracted features. In Figure 2, the plot of Pearson correlation coefficient vs. MIC, it is clear that the absolute values of the Pearson correlation coefficient and MIC are positively correlated. There is only 1 point on the upper left corner of the plot representing a highly correlated pair. It corresponds to *V*_*LV, ES*_ and *EF*_*LV*_, which are of Pearson correlation coefficient −0.80 and MIC 0.51. The strong negative correlation between these 2 features is reasonable, since by definition *EF*_*LV*_ = 1−*V*_*LV, ES*_/*V*_*LV, ED*_, in which *V*_*LV, ED*_ is the LV volume at ED. Therefore, *V*_*LV, ES*_ and *EF*_*LV*_ appear to be redundant. Hence, we exclude *EF*_*LV*_ and select the remaining 8 features for cluster analysis (Table 1).

### 4.3. Cluster Analysis

#### 4.3.1. Gaussian Mixture Model Selection

**Figure 3:**
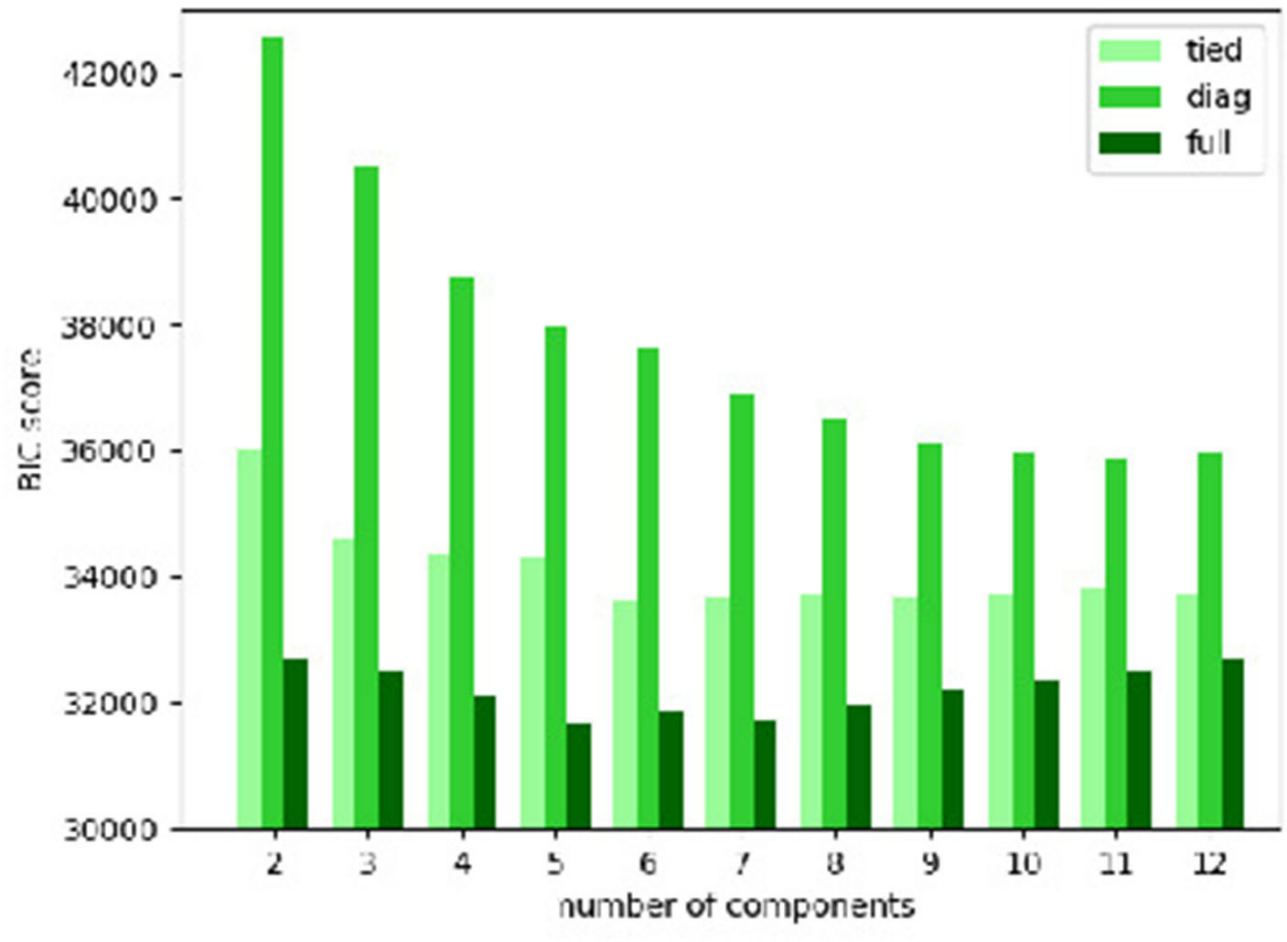
Bayesian information criterion (BIC) scores of Gaussian mixture models with various types of covariance matrix and numbers of components.

The BIC scores of the Gaussian mixture models with various types of covariance matrix and numbers of components are plotted in Figure 3. It is clear that the “full” covariance matrix type is the best among the 3. The “full” covariance matrix type is hence selected.

And in terms of the number of components, the Gaussian mixture models with the “full” covariance matrix type of 3–10 components have the smallest BIC scores. Among them, we find that:

- The models of 3–6 components only generate large clusters, each of which contains at least about 100 cases;
- The models of 7 and 8 components bring about only 1 small cluster (less than a dozen cases);
- The models of 9 and 10 components give rise to 2 small clusters (less than a dozen cases).

According to the statistics^4^ provided by the British Heart Foundation , about 7 million people in the UK are living with cardiovascular diseases, which is more than 10% of the total population. More specifically, if we look at the most common cardiovascular disease categories, the percentages of UK population living with myocardial infarction, atrial fibrillation, and heart failure are about 1.5, 2.0, and 1.4%, respectively. This means that most of the cases in the general population do not have a cardiac pathology. Taking the “healthy volunteer” selection bias of UK Biobank, mentioned in section 2.1, into account, the cases of cardiovascular diseases are hence probably exceedingly rare in UK Biobank. Thus, if there is any cluster that is related to a specific pathological category in an interpretable manner, its size should be small, say, no more than 76 (2% of the 3,822 UK Biobank cases).

So we can now suggest that a component number of 9 or 10 is probably most suitable. We choose the model of 9 components for further analysis. But we would like to point out that the 2 resulting small clusters of the models of 9 and 10 components are very similar in terms of size and cases. So the results and the conclusions shown below will be roughly the same if we use the model of 10 components.

To summarize, the Gaussian mixture model with the “full” covariance matrix type and 9 components is selected.

#### 4.3.2. Analysis of the Resulting Clusters

Among the 9 resulting clusters (termed clusters #1–#9) of the selected model, 2 are of small sizes (clusters #5 and #8). We find that they actually correspond to 2 pathological categories according to the definition given by the ACDC (RVA and DCM, respectively).

**Figure 4:**
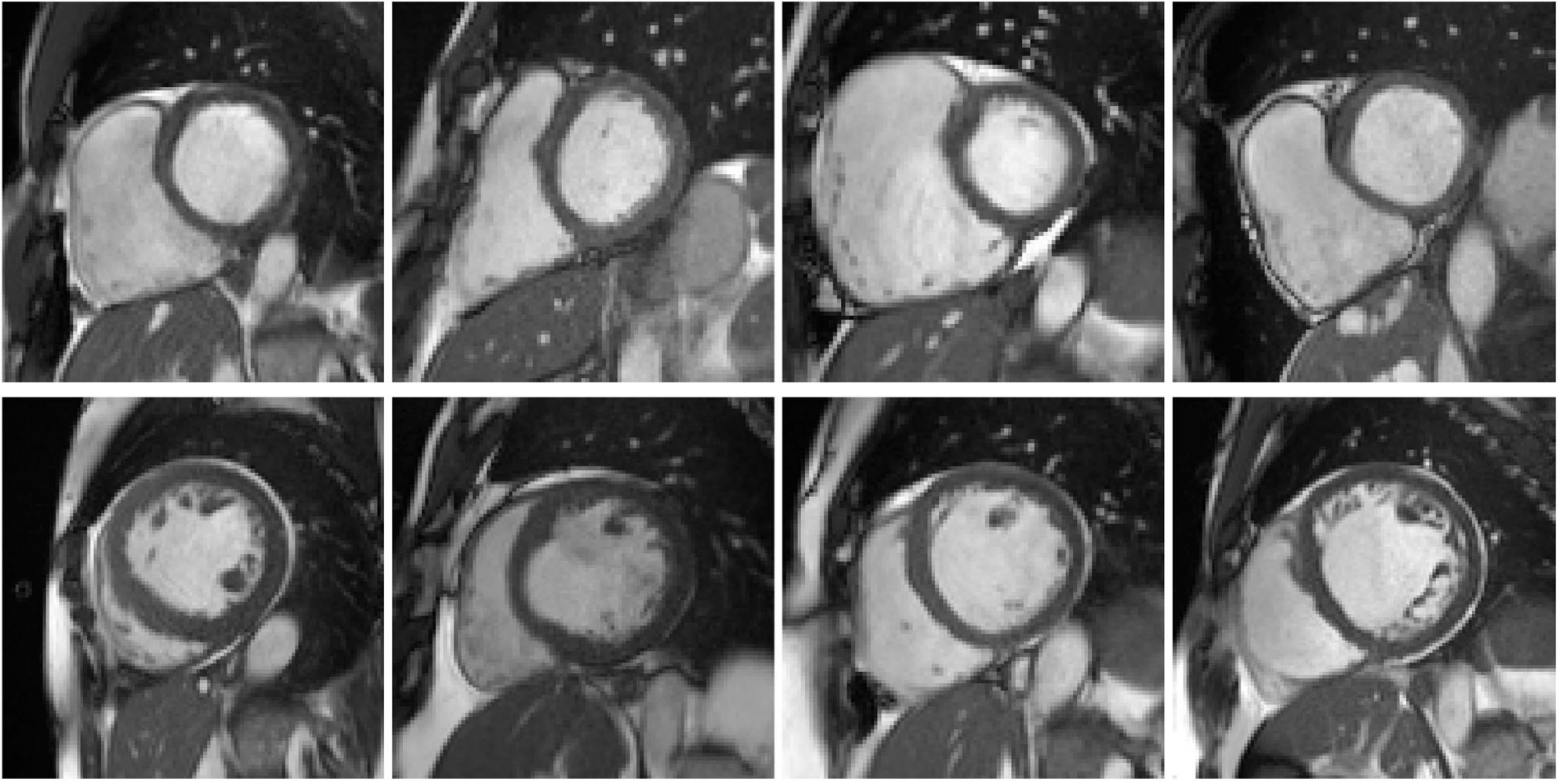
Examples of the cases in clusters #5 and #8. First row: example cases in cluster #5, of which the right ventricles (RVs) appear to be exceptionally large. Second row: cases in cluster #8, of which the left ventricles (LVs) seem to be dilated.

Cluster #5 has 11 cases (examples are given in Figure 4). As listed in Table 2, these cases have exceptionally large RVs, which are above 130 *mL*/*m*^2^. In the ACDC, the RVA cases are described as of RV volumes higher than 110 *mL*/*m*^2^ or RV ejection fraction lower than 40%. Hence according to the definition of ACDC, cluster #5 is a group of cases belonging to RVA.

**Table 2:** Right ventricle (RV) volumes and ejection fraction at end-diastole (ED) of the cases of cluster #5 based on our feature extraction method.

**ID**	**RV volume at ED** (*mL*/*m*^**2**^)	**RV ejection fraction**
2512949	133.13	63.61%
2628396	175.77	43.91%
3423847	140.50	65.24%
3713328	169.65	71.59%
3874816	183.96	56.22%
4366978	134.68	52.53%
4681487	139.82	54.39%
4710306	144.86	29.69%
5101726	145.93	43.82%
5319688	151.30	51.93%
5561149	180.48	41.88%

Cluster #8 has 4 cases (examples are given in Figure 4). As shown in Table 3, these cases have large LV volumes at ED (above 130 *mL*/*m*^2^) and low LV ejection fractions (below 30%). In the ACDC, DCM cases are those with LV volumes larger than 100 *mL*/*m*^2^ and LV ejection fraction lower than 40%. So cluster #8 is a group of DCM cases according to ACDC. In addition, we find that the ground-truth measures of LV volume at ED and LV ejection fraction are available for all 4 cases in UK Biobank (last 2 columns in Table 3). It is straightforward to see in Table 3 that the measures generated by our feature extraction method are quite close to the ground truth.

**Table 3:** Left ventricle (LV) volumes at end-diastole (ED) and ejection fraction of the cases of cluster #8 based on our feature extraction method (the second and third columns).

**ID**	**LV volume at ED (*mL*/*m*^**2**^)**	**LV ejection fraction (%)**	**Ground-truth LV volume at ED (*mL*/*m*^**2**^)**	**Ground-truth LV ejection fraction (%)**
2432774	189.28	19.74	208.24	20
3378112	213.28	18.75	213.03	15
4879002	133.09	27.03	144.59	29
5618713	192.87	26.74	192.43	27

*The same measures provided by the UK Biobank dataset are also shown (the fourth and fifth columns). The 2 sets of measures are quite close to each other*.

For the other 7 clusters, which are of much larger sizes (above 70), we do not identify any clear correspondence between them and the pathological categories defined in the ACDC. This is somewhat expected as the participants of the UK Biobank dataset are from the general population. So most of them are actually healthy. Moreover, only 5 out of many pathological categories are taken into account in our analysis. It is hence not surprising that only 2 clusters are identified as pathological.

### 4.4. Further Analysis for Confirmation

To further confirm the discovered correspondence between the 2 small clusters and the 2 pathological categories, as well as to verify whether the large clusters represent normal cases, in addition to manual verification of the segmentation masks and apparent flow maps to ensure the exactness of the features, we also conduct the following analysis.

#### 4.4.1. Interpretation of the Results of an ACDC Classification Model

We apply a pathology classification model (14) trained using the ACDC dataset on the cases of clusters #5 and #8.

Seven of the 11 cases of cluster #5 are predicted to be RVA, which is as expected. However, the other 4 cases (2512949, 3423847, 4681487, and 5319688) are predicted to be NOR (i.e., normal). We suggest that this is partially due to the difference in the distributions of RV ejection fraction. In ACDC, a great majority of the RVA cases are of RV ejection fraction well below 50%. So the trained model has learned to rely on this feature to determine RVA cases. Yet in UK Biobank, some RVA cases, including the 4 listed above, are of RV ejection fraction above 50%. They are not as severe cases as in ACDC.

All 4 cases of cluster #8 are predicted to be DCM by the classification model, which supports the correspondence between cluster #8 and DCM. In addition, by manually checking the motion, we can confirm areas of hypokinesia and akinesia for these cases but also dyskinesia for 1 case (3378112). For case ID 2432774, we also observe discoordinate movement of the LV myocardium suggestive of bundle branch block, which is a type of electrical conduction disease commonly associated with structural heart disease and heart failure. These observations suggest that these cases might also have some relation to MINF. In fact, as pointed out in the ACDC, the increase in LV volume can be a consequence of the adaptation of LV due to MINF (also called cardiac remodeling).

#### 4.4.2. Reduced Dimensionality Visualization Using Principal Component Analysis

**Figure 5:**
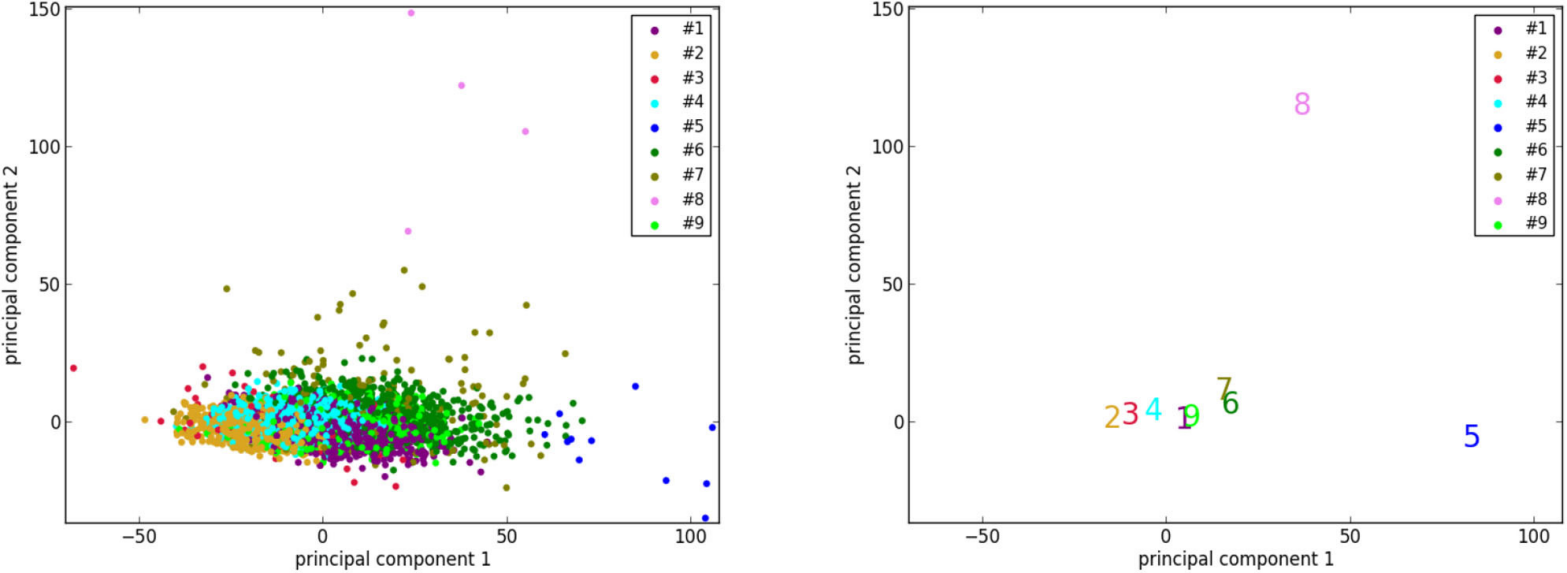
The results of dimensionality reduction by principal component analysis. **(Left)** The data points of the 3,822 UK Biobank cases projected to the space of the 2 principal components. Each data point is colored according to its cluster. **(Right)** Projection of the centers (marked by the corresponding indexes and colors) of the 9 clusters to the same space.

To better visualize the 2 isolated clusters (#5 and #8), we perform a principal component analysis to reduce the dimensionality of the 3,822 vectors of size 8 (8 selected features of 3,822 cases) of UK Biobank to 2. Furthermore, the centers of the 9 clusters are also projected to the sample space of the 2 principal components. As can be seen in Figure 5, the points corresponding to the cases of clusters #5 and #8, as well as the centers of the 2 clusters, are indeed located far away from most of the other points. This supports the suggestion that the cases in clusters #5 and #8, which are pathological, are quite different from most of the cases in the general population.

#### 4.4.3. Visualization Using t-SNE

**Figure 6:**
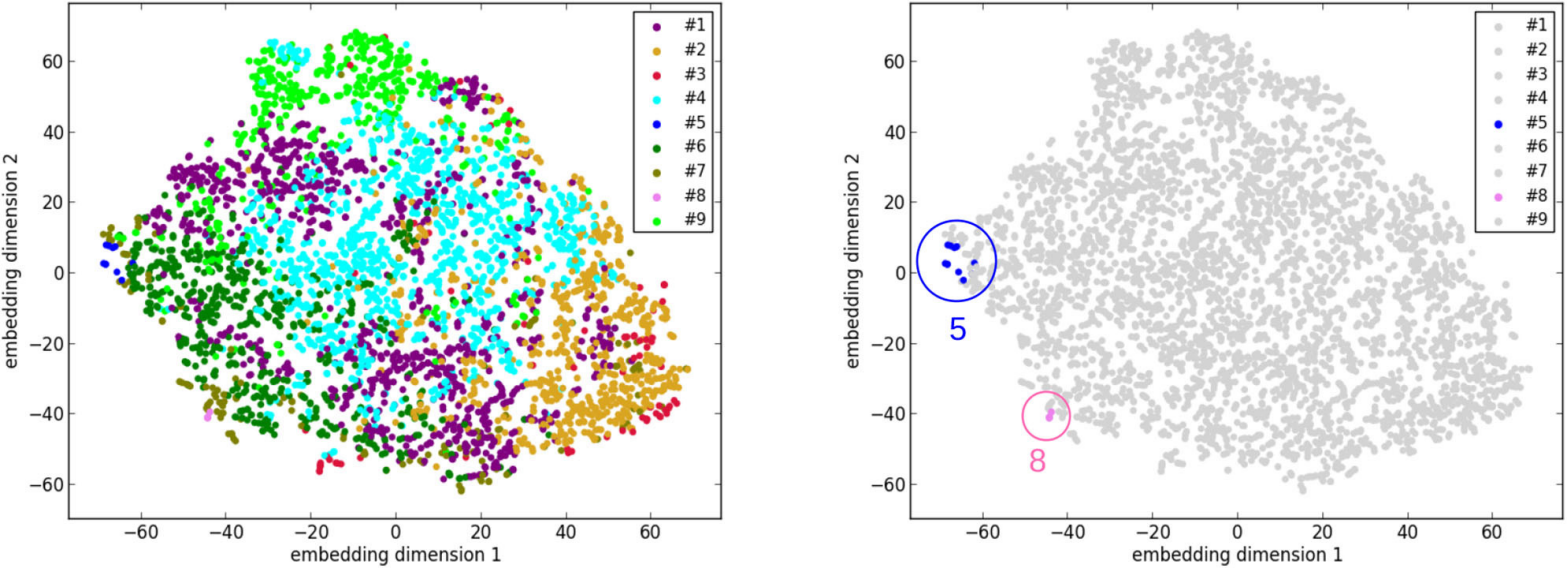
The results of dimensionality reduction by t-distributed stochastic neighbor embedding (t-SNE). **(Left)** The data points of the 3,822 UK Biobank cases in the space of the 2 embedding dimensions after t-SNE. Each data point is colored according to its cluster. **(Right)** A plot similar to the left one with only differences on coloring. Only the points of clusters #5 and #8 are highlighted with colors and circles.

Similarly, another tool to visualize high-dimensional data called t-distributed stochastic neighbor embedding [t-SNE; (36)] is applied. Its main advantage is the ability to preserve local structure. So roughly speaking, points which are close to one another in the high-dimensional space will still be close to one another after the dimensionality reduction. t-SNE is applied to the set of the 3,822 vectors of the UK Biobank cases, as well as to the set of 3,831 vectors that consists of the 3,822 UK Biobank cases and the 9 cluster centers. Before applying t-SNE, a normalization is performed for each feature of the original data. The purpose is to make sure that each feature is on the same scale and hence has the same importance in t-SNE. As shown in Figure 6, the points of the cases and the centers of clusters #5 and #8 are at the edge of the ensemble of points in the embedding space. This phenomenon is again consistent with the suggestion that clusters #5 and #8 correspond to pathological cases, which are rather different from the other cases in the general population.

#### 4.4.4. Examination of the Two Largest Clusters

As pointed out previously, while the pathological categories of clusters #5 and #8 are identifiable, we do not see how the other 7 large clusters correspond to any cardiac pathology. In particular, the largest clusters which are of several hundreds or even more cases probably represent groups of normal cases. To verify this, we further examine the 2 largest clusters (#1 and #4, 889 and 1,075 cases, respectively).

**Figure 7:**
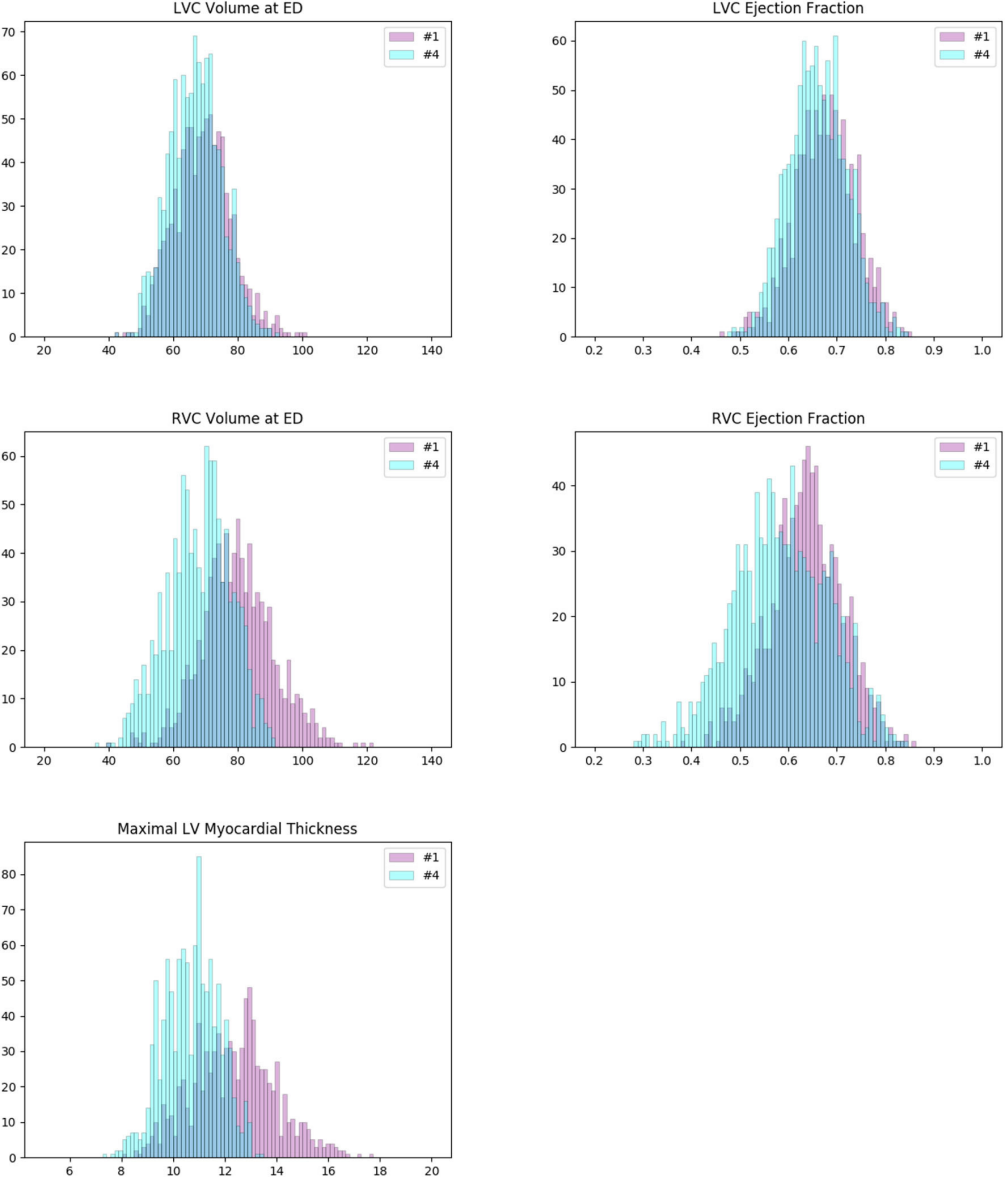
Histograms of some important measures of the cases in clusters #1 (pink) and #4 (cyan). The colors of the columns are set to be partially transparent such that their overlaps appear to be dark blue. The distributions of #1 and #4 are pretty similar in terms of left ventricle (LV) volume and LV ejection fraction (first row). But they are different in case of right ventricle (RV) volume, RV ejection fraction, and maximal myocardial thickness (second and third rows). On average, the cases of #1 have larger RVs with higher ejection fractions. And their myocardiums also tend to be thicker than that of the cases of #4. For both clusters, the measures are well in normal ranges according to the definitions given by Automatic Cardiac Diagnosis Challenge (ACDC).

We plot the histograms of their ventricle volumes and ejection fractions, as well as their maximal myocardial thicknesses (Figure 7). The distributions of #1 and #4 look pretty similar in terms of LV volume and LV ejection fraction. But they are different on RV volume, RV ejection fraction and maximal myocardial thickness. On average, the cases of #4 have larger RVs with higher ejection fractions. And their myocardiums also tend to be thicker than that of the cases of #1. Furthermore, we perform the unpaired unequal variance *t*-test to prove that the corresponding means of the distributions of #1 and #4 are different. Under the null hypotheses that the corresponding distributions have the same mean, the *p*-values for LV volume, LV ejection fraction, RV volume, RV ejection fraction and maximal myocardial thickness are all much below 0.05 (<10^−7^), which are small enough to reject the null hypotheses. This means that clusters #1 and #4 actually exhibit significant different values of the 5 features (LV volume at ED, LV ejection fraction, RV volume at ED, RV ejection fraction and maximal myocardial thickness).

For both clusters, at least a great majority of the cases satisfy:

- LV volumes at ED less than 100 *mL*/*m*^2^;
- LV ejection fraction above 40%;
- RV volumes at ED less than 110 *mL*/*m*^2^;
- RV ejection fraction above 40%;
- Maximal myocardial thickness less than 15 mm.

Hence according to the definitions in ACDC, these 2 clusters do not correspond to any of the 4 pathological categories (DCM, HCM, MINF, and RVA).

#### 4.4.5. Examination of the Seven Large Clusters

To further understand the 7 large clusters, we first systematically perform the unpaired unequal variance *t*-test. For each pair of clusters in the 7 large clusters, and for each of the 8 extracted features, under the null hypothesis the distribution of the feature has the same mean for both clusters, and the *p*-value is computed. In this way, 21 × 8 = 168 *p*-values are obtained. In total, 149 *p*-values among them are below 0.05, which are small enough to reject the corresponding null hypotheses. This confirms that the clusters have different distributions on the features. Nineteen *p*-values among them are above 0.05, which signify a kind of similarity between pairs of clusters (Table 4). Similarly, we perform the unpaired 2-sided Mann-Whitney rank tests, under the null hypotheses that the corresponding distributions of the features are the same for both clusters. And we find again that a great majority (147) of the *p*-values are below 0.05 such that the corresponding null hypotheses can be rejected.

**Table 4:** The large *p*-values of the unpaired unequal variance *t*-tests for the 21 pairs of clusters in the 7 large clusters, and for the 8 extracted features, under the null hypothesis that the distribution of the feature has the same mean for both clusters.

**Cluster pair**	***p*-values above 0.05 (and the corresponding features)**
(#1, #4)	0.07 (*V*_*LV, ES*_)
(#1, #6)	0.56 (*RMD*), 0.05 (*TMD*)
(#1, #9)	0.55 (*V*_*RV, ED*_), 0.76 (*R*_*RVLV, ED*_)
(#2, #3)	0.17 (*R*_*RVLV, ED*_), 0.80 (*R*_*LVMLV, ED*_)
(#2, #4)	0.31 (*TMD*)
(#2, #7)	0.85 (*R*_*RVLV, ED*_), 0.76 (*RMD*)
(#3, #4)	0.29 (*R*_*RVLV, ED*_)
(#3, #6)	0.12 (*EF*_*RV*_)
(#3, #7)	0.07 (*EF*_*RV*_), 0.28 (*R*_*RVLV, ED*_), 0.61 (*MT*_*LVM, ED*_), 0.25 (*TMD*)
(#4, #6)	0.70 (*R*_*LVMLV, ED*_), 0.14 (*TMD*)
(#6, #7)	0.27 (*EF*_*RV*_)

*For most of the cluster pairs and features, the p-values are below 0.05*.

#### 4.4.6. Measures by the Automatic Pipeline vs. the Ground Truth

As mentioned previously, for part of the UK Biobank cases, the ground-truth measures given by the InlineVF analysis algorithm of LV volumes at ED and ES and LV ejection fraction are available. In particular, among the 3,822 cases used in this paper, we have access to all of the 3 ground-truth measures for 3,212 cases. The comparison between the means and standard deviations of the measures generated by the automatic pipeline used in this paper and the ground-truth measures are shown in Table 5. It is clear that the ground-truth measures of the volumes are higher and of larger standard deviations than those estimated by the automatic pipeline.

**Table 5:** The means and standard deviations of the measures (in *mL*/*m*^2^) by the automatic pipeline vs. the ground truth.

	**Automatic pipeline**	**Ground truth**
LV volume at ED (*mL*/*m*^2^)	70.56 (13.91)	75.48 (28.62)
LV volume at ES (*mL*/*m*^2^)	24.06 (9.02)	33.87 (22.82)
LV ejection fraction	66.41% (7.33%)	56.04% (6.53%)

**Figure 8:**
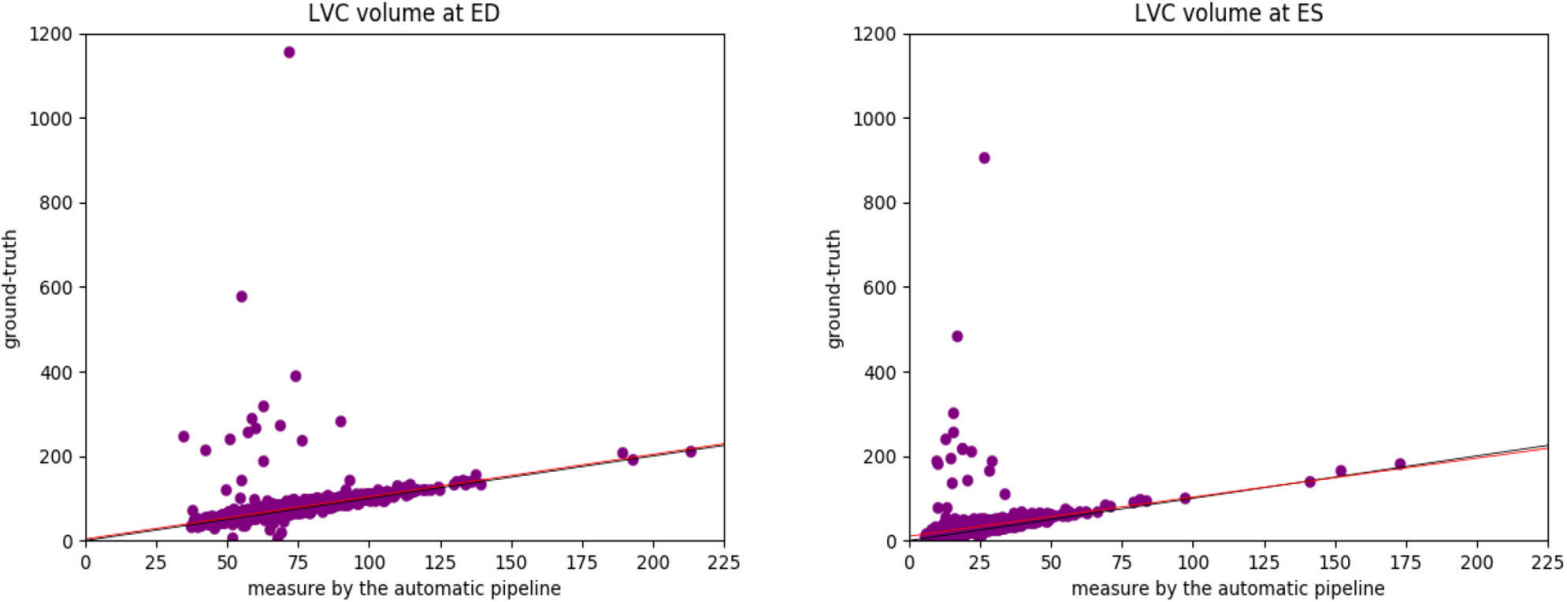
The plots of the measures (in *mL*/*m*^2^) generated by the automatic pipeline against the ground truth for the LV volume at ED **(Left)** and at ES **(Right)**. We can see that the ground-truth values contain some obvious outliers, which are often of values well above the realistic range of LV volumes. This explains the fact that the ground-truth volumes have higher means and larger standard deviations than those estimated by the automatic pipeline. The lines corresponding to the robust linear regression models (red) and the lines corresponding to *ground-truth*=*automatic-pipeline* (black) are also plotted. The red line and the black line almost overlap with each other.

To better understand the cause of these differences, we plot the points of the measures in Figure 8. We can see that the ground-truth values contain some obvious outliers, which are often of values well above the realistic range of LV volumes. This explains the fact that the ground-truth volumes have higher means and larger standard deviations than those estimated by the automatic pipeline. Moreover, proportionally, the mean of the ground-truth values of LV volume at ED is 7.0% (= 75.48/70.56 - 1) above that of the estimates by the automatic pipeline, while for LV volume at ES the ground-truth is on average 40.8% (= 33.87/24.06 -1) higher than the values obtained via the automatic pipeline. This also explains why the ground truth of LV ejection fraction is on average lower than that given by the automatic pipeline. The models obtained by the robust linear regression using Huber's criterion for LV volume at ED and ES are *ground-truth*=*1.002*×*automatic-pipeline+3.373* and *ground-truth=0.923*×*automatic-pipeline+10.303*, respectively. The lines corresponding to the robust linear regression models (red) and the lines corresponding to *ground-truth=automatic-pipeline* (black) are plotted in Figure 8. On both graphs in Figure 8, the red line and the black line almost overlap with each other. This means that our regression lines are near the lines of identity, which signifies a similarity between the measures by our method and those based on the InlineVF algorithm. By comparing the regression lines and identity lines in Figure 4 of (37), we can also conclude a similarity between the measures derived from manual segmentation and those based on the InlineVF algorithm. Hence, our method actually generates measures that are close to both manual and InlineVF values.

We believe that the differences between the measures by the automatic pipeline used in this paper and the ground truth are partially due to the lack of quality control on the ground truth. In fact, as pointed out in (37), the ground truth is generated by the InlineVF algorithm, which may fail and hence make unreliable predictions on some cases. Without quality control, these failures causes the outliers in Figure 8.

Also, in addition to being useful for quality control, methods using ground truth like the linear regression performed above are complementary to unsupervised learning methods. Actually, in our method, ground truth is necessary for training the neural networks, which are then used for feature extraction. The combination of supervised and unsupervised methods hence looks worth further exploration.

## 5. Conclusion and Discussion

In this paper, we proposed a method of unsupervised cluster analysis on a large unlabeled dataset (UK Biobank) of the general population to identify pathological cases based on shape-related and motion-characteristic features extracted from cardiac cine MRI images. As far as we know, this is a topic that has rarely been studied before. In our cluster analysis, a Gaussian mixture model is applied to cluster similar cases together without supervision. As a result, among the generated clusters, we identify 2 that probably correspond to 2 cardiac pathological categories. This idea is further supported by the observations on the results of a trained classification model and of the dimensionality reduction tools including principal component analysis and t-SNE.

As more and more large and unlabeled datasets are available in the community, researchers will be able to extract interesting information by data mining. Identification of cardiac pathology is just one among other topics such as the analysis of motion patterns, the relationship between motion and shape features, and so on. In the future, more research may be carried out by including more data and different types of data (38), using more features, targeting other abnormalities or phenotype properties, etc. For instance, 1 main advantage of cardiac MRI is that it allows tissue characterization with late gadolinium enhancement and parametric maps (T1, T2, T2*). As a result, more useful features might be extracted from these variables for analysis. Furthermore, if feasible, using information from a 4-chamber segmentation (both ventricles and both atria) might improve the performance of pathology detection. Various unsupervised learning methods (39) other than a Gaussian mixture model can also be applied.

## Data Availability Statement

The two datasets analyzed in this study was obtained from UK Biobank under application 2964 and Automatic Cardiac Diagnosis Challenge (ACDC), respectively.

## Author Contributions

All authors contributed to the design and implementation of the research, the analysis of the results, and the writing of the manuscript.

## Conflict of Interest

SP provided consultant services to Circle Cardiovascular Imaging Inc., Calgary, Alberta, Canada. The remaining authors declare that the research was conducted in the absence of any commercial or financial relationships that could be construed as a potential conflict of interest.

## Footnotes

^1^Application Number 2964.

^2^https://www.ukbiobank.ac.uk/scientists-3/

^3^https://www.creatis.insa-lyon.fr/Challenge/acdc/index.html

^4^https://www.bhf.org.uk/what-we-do/our-research/heart-statistics (accessed November 18, 2019).

## References

[1] Petersen S Aung N Sanghvi M Zemrak F Fung K Paiva J. Reference ranges for cardiac structure and function using cardiovascular magnetic resonance (CMR) in Caucasians from the UK Biobank population cohort. J Cardiovasc Magn Reson. (2017) 19:18. 10.1186/s12968-017-0327-9 28178995 PMC5304550

[2] Attar R Pereanez M Gooya A Alba X Zhang L Piechnik S High throughput computation of reference ranges of biventricular cardiac function on the UK Biobank population cohort. arXiv [Preprint] arXiv:190103326 (2019). 10.1007/978-3-030-12029-0_13

[3] Toga A Crawford K. The Alzheimer's disease neuroimaging initiative informatics core: a decade in review. Alzheimers Dement. (2015) 11:832–9. 10.1016/j.jalz.2015.04.004 26194316 PMC4510464

[4] Rueckert D Glocker B Kainz B. Learning clinically useful information from images: past, present and future. Med Image Anal. (2016) 33:13–8. 10.1016/j.media.2016.06.009 27344105

[5] Suinesiaputra A McCulloch A Nash M Pontre B Young A. Cardiac image modelling: breadth and depth in heart disease. Med Image Anal. (2016) 33:38–43. 10.1016/j.media.2016.06.027 27349830 PMC5123588

[6] Zhang S Metaxas D. Large-scale medical image analytics: recent methodologies, applications and future directions. Med Image Anal. (2016) 33:98–101. 10.1016/j.media.2016.06.010 27503077

[7] Barillot C Edan G Commowick O. Imaging biomarkers in multiple sclerosis: from image analysis to population imaging. Med Image Anal. (2016) 33:134–9. 10.1016/j.media.2016.06.017 27374128

[8] de Bruijne M. Machine learning approaches in medical image analysis: from detection to diagnosis. Med Image Anal. (2016) 33:94–7. 10.1016/j.media.2016.06.032 27481324

[9] Weese J Lorenz C. Four challenges in medical image analysis from an industrial perspective. Med Image Anal. (2016) 33:44–9. 10.1016/j.media.2016.06.023 27344939

[10] Parisot S Ktena S Ferrante E Lee M Guerrero R Glocker B. Disease prediction using graph convolutional networks: application to autism spectrum disorder and Alzheimer's disease. Med Image Anal. (2018) 48:117–30. 10.1016/j.media.2018.06.001 29890408

[11] Liu M Zhang J Nie D Yap P Shen D. Anatomical landmark based deep feature representation for MR images in brain disease diagnosis. IEEE J Biomed Health Inform. (2018) 22:1476–85. 10.1109/JBHI.2018.2791863 29994175 PMC6238951

[12] Madabhushi A Lee G. Image analysis and machine learning in digital pathology: challenges and opportunities. Med Image Anal. (2017) 33:170–5. 10.1016/j.media.2016.06.037 27423409 PMC5556681

[13] Komura D Ishikawa S. Machine learning methods for histopathological image analysis. Comput Struct Biotechnol. (2018) 16:34–42. 10.1016/j.csbj.2018.01.001 30275936 PMC6158771

[14] Zheng Q Delingette H Ayache N. Explainable cardiac pathology classification on cine MRI with motion characterization by semi-supervised learning of apparent flow. arXiv [Preprint] arXiv:181103433 (2018). 10.1016/j.media.2019.06.001 31200290

[15] Khened M Alex V Krishnamurthi G. Fully convolutional multi-scale residual DenseNets for cardiac segmentation and automated cardiac diagnosis using ensemble of classifiers. arXiv [Preprint] arXiv:180105173 (2018). 10.1016/j.media.2018.10.004 30390512

[16] Khened M Alex V Krishnamurthi G Densely connected fully convolutional network for short-axis cardiac cine MR image segmentation and heart diagnosis using random forest. In: Proc. Statistical Atlases and Computational Models of the Heart (STACOM), ACDC challenge, MICCAI'17 Workshop. Quebec City (2017). 10.1007/978-3-319-75541-0_15

[17] Isensee F Jaeger P Full P Wolf I Engelhardt S Maier-Hein K Automatic cardiac disease assessment on cine-MRI via time-series segmentation and domain specific features. In: Proc. Statistical Atlases and Computational Models of the Heart (STACOM), ACDC Challenge, MICCAI'17 Workshop. Quebec City (2017). 10.1007/978-3-319-75541-0_13

[18] Wolterink J Leiner T Viergever M Isgum I Automatic segmentation and disease classification using cardiac cine MR images. In: Proc. Statistical Atlases and Computational Models of the Heart (STACOM), ACDC Challenge, MICCAI'17 Workshop. Quebec City (2017). 10.1007/978-3-319-75541-0_11

[19] Cetin I Sanroma G Petersen S Napel S Camara O Ballester M A radiomics approach to computer-aided diagnosis with cardiac cine-MRI. In: Proc. Statistical Atlases and Computational Models of the Heart (STACOM), ACDC Challenge, MICCAI'17 Workshop (2017). 10.1007/978-3-319-75541-0_9

[20] Dragulescu A Mertens L Friedberg M. Interpretation of left ventricular diastolic dysfunction in children with cardiomyopathy by echocardiography problems and limitations. Circulation. (2013) 6:254–61. 10.1161/CIRCIMAGING.112.000175 23343514

[21] Kinani J Silva A Funes F Vargas D Diaz E Arellano A Medical imaging lesion detection based on unified gravitational fuzzy clustering. J Healthcare Eng. (2017) 2017:1–14. 10.1155/2017/8536206 PMC5660817 29158887

[22] Moriya T Roth H Nakamura S Oda H Nagara K Oda M Unsupervised segmentation of 3D medical images based on clustering and deep representation learning. arXiv [Preprint] arXiv:180403830 (2018). 10.1117/12.2293414

[23] Moldovanu S Obreja C Moraru L Threshold selection for classification of MR brain images by clustering method. in: TIM14 Physics Conference - Physics Without Frontiers. (2015). 10.1063/1.4937257

[24] Kawadiwale R Rane M Clustering techniques for brain tumor detection. In: Conf. on Recent Trends in Information, Telecommunication and Computing, ITC. Chandigarh (2014). p. 299-305.

[25] Zheng Q Delingette H Duchateau N Ayache N. 3D consistent and robust segmentation of cardiac images by deep learning with spatial propagation. IEEE Trans Med Imaging. (2018). 37:2137–48. 10.1109/TMI.2018.2820742 29994087

[26] Petersen S Matthews P Francis J Robson M Zemrak F Boubertakh R. UK Biobank's cardiovascular magnetic resonance protocol. J Cardiovasc Magn Reson. (2016) 18:8. 10.1186/s12968-016-0227-4 26830817 PMC4736703

[27] Jolly M Guetter C Lu X Xue H Guehring J Automatic segmentation of the myocardium in cine MR images using deformable registration. In: Statistical Atlases and Computational Models of the Heart Imaging and Modelling Challenges. Nagoya: Springer City (2013) p. 98–108. 10.1007/978-3-642-28326-0_10

[28] Lu X Georgescu B Jolly M Guehring J Young A Cowan B. Cardiac anchoring in MRI through context modeling. Med Image Comput Comput Assist Interv. (2010) 13:383–90. 10.1007/978-3-642-15705-9_47 20879254

[29] Fry A Littlejohns T Sudlow C Doherty N Adamska L Sprosen T. Comparison of sociodemographic and health-related characteristics of UK Biobank participants with those of the general population. Am J Epidemiol. (2017) 186:1026–34. 10.1093/aje/kwx246 28641372 PMC5860371

[30] Zheng Q Delingette H Duchateau N Ayache N 3D consistent biventricular myocardial segmentation using deep learning for mesh generation. arXiv [Preprint] arXiv:180311080 (2018).

[31] Zheng Q Deep Learning for Robust Segmentation and Explainable Analysis of 3d and Dynamic Cardiac Images. Université Côte d'Azur (2019).

[32] Reshed D Reshef Y Finucane H Grossman S McVean G Turnbaugh P. Detecting novel associations in large data sets. Science. (2011) 334:1518–24. 10.1126/science.1205438 22174245 PMC3325791

[33] Reynolds D Gaussian mixture models. In: Encyclopedia of Biometrics. Springer City (2009) p. 659-663. 10.1007/978-0-387-73003-5_196

[34] Pedregosa F Varoquaux G Gramfort A Michel V Thirion B Grisel O Scikit-learn: machine learning in Python. J Mach Learn Res. (2011) 12:2825–30.

[35] Wit E van den Heuvel E Romeijn J ‘All models are wrong.': an introduction to model uncertainty. Statistica Neerlandica. (2012) 66:217–36. 10.1111/j.1467-9574.2012.00530.x

[36] van der Maaten L Hinton G Visualizing data using t-sne. J Mach Learn Res. (2008) 9:2579–605.

[37] Suinesiaputra A Sanghvi M Aung N Paiva J Zemrak F Fung K. Fully-automated left ventricular mass and volume MRI analysis in the UK Biobank population cohort: evaluation of initial results. Int J Cardiovasc Imaging. (2018) 34:281–91. 10.1007/s10554-017-1225-9 28836039 PMC5809564

[38] Kohli M Summers R Geis J. Medical image data and datasets in the era of machine learning-whitepaper from the 2016. C-MIMI meeting dataset session. J Digit Imaging. (2017) 30:392–9. 10.1007/s10278-017-9976-3 28516233 PMC5537092

[39] Raza K Singh N A tour of unsupervised deep learning for medical image analysis. arXiv [Preprint] arXiv:181207715 (2018). 10.2174/1573405617666210127154257 33504314

[40] Zheng Q Delingette H Fung K Petersen S Ayache N Unsupervised shape and motion analysis of 3822 cardiac 4D MRIs of UK Biobank. arXiv [Preprint] arXiv:190205811 (2019).

